# The Efficacy, Tolerance, and Adverse Events of Endoscopic Intragastric Balloon in Obese and Overweight Patients: A Retrospective Cross-Sectional Study

**DOI:** 10.7759/cureus.56528

**Published:** 2024-03-20

**Authors:** Mohamed W Mohamed, Noora R Alkhater, Faisal O Abubaker, Omar E Sharif

**Affiliations:** 1 Internal Medicine, King Hamad University Hospital, Muharraq, BHR; 2 Gastroenterology and Hepatology, King Hamad University Hospital, Muharraq, BHR

**Keywords:** intragastric balloon, overweight, endoscopy, obesity, weight loss

## Abstract

Introduction

Obesity is a pandemic causing a significant burden on healthcare systems and carries increased morbidity and mortality. One of the options for managing obesity is endoscopic intragastric balloon (IGB) insertion. The aim of the study is to assess the efficacy, tolerance, and side effects of IGB insertion in overweight and obese patients.

Methods

This is a cross-sectional retrospective study that includes 71 patients who underwent IGB insertion from 2015 to 2019 at King Hamad University Hospital (KHUH), Kingdom of Bahrain. Records of these patients were accessed to assess the percentage of weight loss at the time of balloon removal, complications, and tolerance of the procedure. Furthermore, telephonic interviews were conducted to enquire about side effects and the satisfaction of the procedure.

Results

A total of 57 patients were included in the weight loss analysis. Thirteen patients did not tolerate the balloon, and one patient had a balloon rupture. The patients experienced a significant reduction in weight upon balloon removal with a mean of 9.74 ± 8.71 kg (p-value of <0.001) and percentage total body weight loss of 10.48 ± 8.07 (p-value of <0.001). A significant reduction was also seen in the body mass index of 3.67 ± 3.57 (p-value of <0.001). The most frequent side effects were nausea, vomiting, and abdominal pain. No major complications or mortalities occurred.

Conclusion

Intragastric balloons are effective in establishing weight loss. Among patients who tolerated the procedure, the most frequently reported side effects were nausea, vomiting, and abdominal pain.

## Introduction

Obesity is a disease that continued to rise until it became a pandemic worldwide [1]. In 2016, the estimated number of adults who were obese or overweight was approaching two billion globally. Since 1975, the rate of obesity in the Kingdom of Bahrain has doubled [1]. Furthermore, according to the National Health Survey of Bahrain that was conducted in 2018, the prevalence of obesity in the Kingdom of Bahrain in those more than 18 years old was 33% in males and 42.5% in females [2].

Obesity is associated with a variety of health-related problems and comorbidities, including hypertension, diabetes mellitus, dyslipidemia, coronary heart disease, stroke, sleep apnea, osteoarthritis, gallbladder disease, gastroesophageal reflux disease (GERD), nonalcoholic fatty liver disease (NAFLD), and cancer. Furthermore, it is associated with an increased risk of all-cause and cardiovascular mortality [3,4].

The spectrum of obesity management traditionally is known to begin with lifestyle modification, including diet and exercise then pharmacotherapy [5,6]. Bariatric surgery was the only option for those patients who failed medical therapy. In recent years, with the innovation and revolution that occurred in endoscopic procedures, several ways of treatments have been introduced to the world of obesity management. Primary endoscopic therapy includes two main types: gastric and small bowel interventions. Gastric interventions are composed of space-occupying therapy, gastric remodeling technique, and aspiration therapy [7]. On the other hand, small bowel interventions include endobarrier sleeve, duodenal mucosal resurfacing, endoluminal bypass, and incisionless anastomosis system [7].

Bariatric surgeries are considered the most efficient way for remarkable weight loss. However, it is associated with a higher risk of morbidity and mortality [8]. Intragastric balloon (IGB) therapy is an option for overweight or obese patients with a body mass index (BMI) of greater than 27 kg/m^2^ in Europe or 30 kg/m^2^ in the United States (US) who have failed previous attempts at weight management with diet and exercise alone [9]. IGB insertion can also be used as an early intervention for patients up to the BMI of 35 kg/m^2^ for those who have a higher BMI but refuse surgery or as a bridge to bariatric surgery [6,10-12].

The aim of the study is to assess the efficacy, tolerance, and side effects of IGB in overweight and obese patients. The objectives of the study are to assess the mean total body weight loss (TBWL), mean percentage TBWL, mean percentage excess body weight loss (EBWL), and body mass index (BMI) reduction after IGB insertion and to assess the side effects and the patient’s tolerance and satisfaction.

## Materials and methods

This retrospective cross-sectional study was conducted at the endoscopy unit at King Hamad University Hospital (KHUH), a tertiary care university hospital in the Kingdom of Bahrain. All patients attending KHUH from 2015 to 2019 for endoscopic IGB insertion were included in the study, with a total of 71 patients. Inclusion criteria included all patients aged 18-65 years old with a BMI above 25 kg/m² who were eligible for IGB insertion and had the balloon removed after six months post insertion. Exclusion criteria included patients with large hiatal hernia (>5 cm), active duodenal or gastric ulcer, gastrointestinal neoplasm, and active gastrointestinal bleeding or patients who lost follow-up before removal of the IGB.

Endoscopy

The IGBs that were used are Medsil (JSC MedSil, Russia) and Medicone (Medicone, Brazil). These balloons are saline-filled balloons and can be filled with a maximum volume of 700 mL. For 12 patients, the type of balloon used was not mentioned or they had another type of balloon inserted.

Balloons were inserted endoscopically in an outpatient setting after the administration of sedatives, such as midazolam and fentanyl intravenously. In the beginning, diagnostic gastroduodenoscopy was performed to rule out any contraindications. Then, the balloon was implanted under direct endoscopic visualization. Balloons were filled with saline accompanied with methylene blue (MB). MB was added as it would stain the urine blue if there was any leakage or rupture in IGB, improving the detection of complications. After insertion, the patients stayed in the recovery room for almost two hours. Upon discharge, the patients were prescribed antiemetics, such as metoclopramide or domperidone, ondansetron, and proton pump inhibitor (PPI) for 7-10 days. Then, the PPI was continued till balloon extraction [13].

Data collection and measurements

The records of the endoscopy unit at KHUH were reviewed to obtain the list of patients who underwent IGB insertion between the years 2015 and 2019. Data collected included dates of insertion and removal of the IGB, whether the patient tolerated the gastric balloon or not, weight of the patient on insertion and removal, and complications. The patients who did not tolerate the IGB were excluded from the weight loss analysis. IGB intolerance was defined as the removal of the IGB prior to the recommended period of six months due to side effects. 

To facilitate the maximum participation rate, a telephonic interview was conducted between the investigators and patients. The interviews were conducted in 2019. After agreeing to participate in the research, they were asked a set of questions, and the questionnaire was filled out by the investigator conducting the phone calls. These questions assessed several key points, including the side effect profile, satisfaction from the procedure (yes = satisfied, no = not satisfied), and the need for a subsequent bariatric surgery. Of the 71 patients who were contacted, 65 patients responded to the telephonic interviews conducted. The remaining six patients either did not answer the phone or had incorrect phone numbers listed.

Excess body weight (EBW) was calculated as EBW (kg) = weight - ideal body weight (IBW). IBW was calculated via the Devine formula [14].

Ethics

Ethical approval was obtained from the institutional review board (IRB) of KHUH (reference number 19-294). Verbal consent was taken from the participants in the study.

Statistics

Data collected were analyzed via IBM SPSS Statistics for Windows, version 25 (released 2017; IBM Corp., Armonk, New York, United States), and variables, like TBWL, BMI before and after IGB insertion, mean %EBWL, mean percentage TBWL, tolerance, and side effects, were assessed. Descriptive statistics was used for computing the frequencies, means, and percentages. Continuous variables were analyzed using Student's t-test. Nonparametric tests were used wherever applicable.

## Results

**Figure 1:**
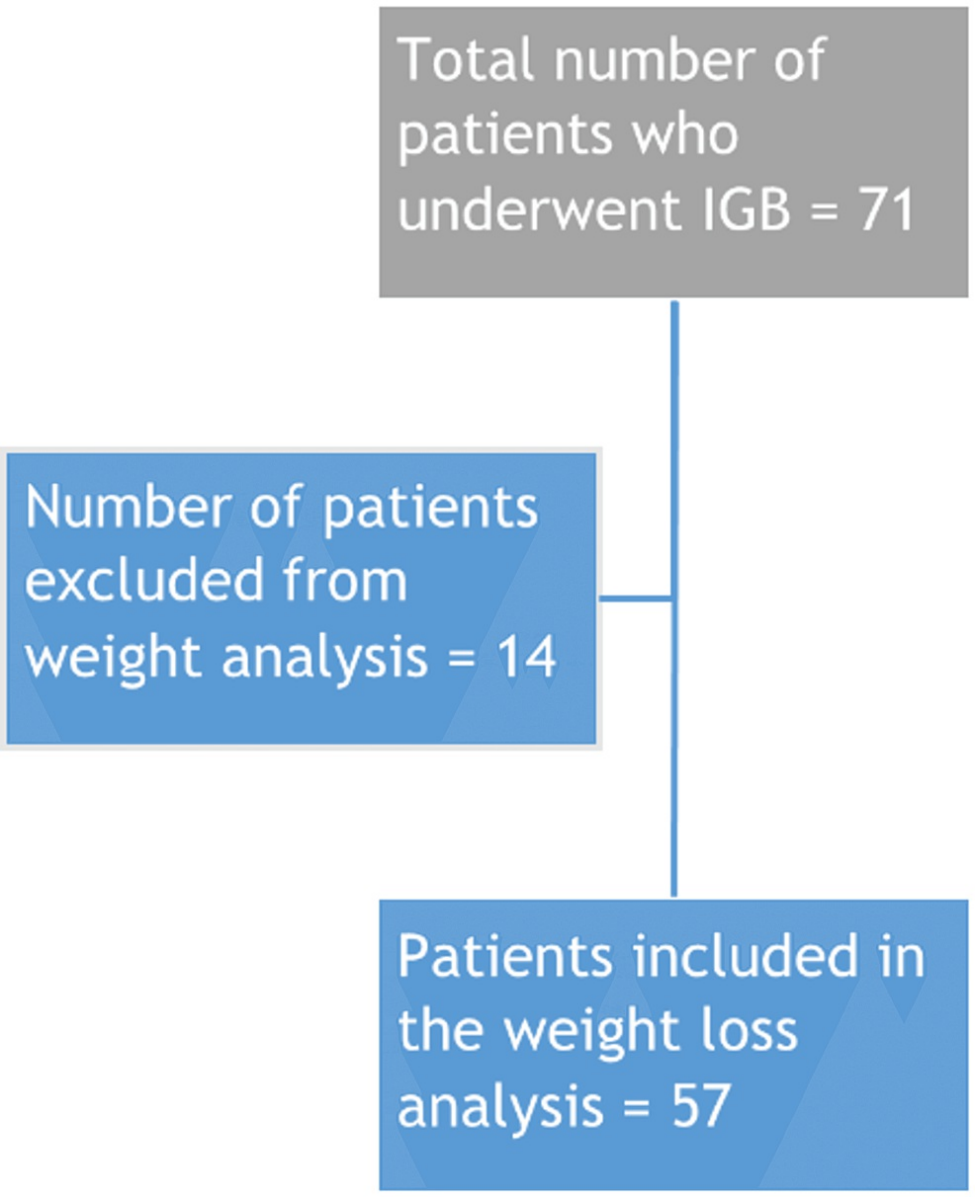
Patient enrollment Flow chart of patient enrollment and number of patients included in the weight loss analysis.

The total number of patients enrolled in the study was 71 patients, of which 14 were excluded from the weight loss analysis (Figure 1) as 13 patients did not tolerate the balloon due to intractable side effects, with subsequent premature removal of the balloon (all 13 patients removed the balloon within around two weeks of insertion) while one patient had a ruptured balloon at around four months after insertion. The baseline characteristics of the patients and frequency of comorbidities are noted in Table 1.

**Table 1:** Baseline characteristics of the study participants IGB: intragastric balloon

Variable	Value
Average age (years)	35.08 ± 9.69
Average body weight (Kg)	98.57 ± 21.2
Average BMI (kg/m^2^)	36.30 ± 6.08
Average excess weight (Kg)	40.92 ± 17.03
Average duration of IGB (months)	6.96 ± 2.053
Diabetes mellitus	1 (2%)
Hypertension	3 (6%)
Dyslipidemia	2 (4%)
Ischemic heart disease	None

Fifty-seven patients were included in the weight loss analysis and completed at least the six-month period with the IGB in place, 43 (75%) of which were females and 14 (25%) were males.

Eight of the 57 patients had their IGB removed beyond the recommended six months post insertion; five patients removed the IGB at eight to 10 months post insertion with no significant complications at the time of removal. However, three patients had complications, including balloon rupture, migration, and passage in the stool.

It was also noted that two patients underwent IGB insertion twice to achieve further weight reduction.

Overall, the patients experienced a significant reduction in weight at the time of removal of IGB from an average initial weight of 98.57 ± 21.2 to 88.82 ± 22.4 kg with a mean difference of 9.74 ± 8.71 kg (p-value of <0.001). A significant reduction was also seen in the BMI from an average baseline of 36.3 kg/m^2^ ± 6.1 to 32.68 kg/m^2^ ± 6.9 with a mean difference of 3.67 kg/m^2^ (p-value of <0.001). The mean percentage TBWL was 10.48 % ±8.07 (p-value <0.001), while the mean %EBWL was 27.21 kg ± 20.72 (p-value <0.001; Table 2).

**Table 2:** Weight loss data of the participants

Variable	Result	P value
Mean total body weight lost (Kg)	9.74 ± 8.71	<0.001
Mean reduction in BMI	3.67 ± 3.57	<0.001
Mean percentage total body weight lost (%)	10.48 ± 8.07	<0.001
Mean percentage excess body weight lost (%)	27.21 ± 20.72	<0.001

**Figure 2:**
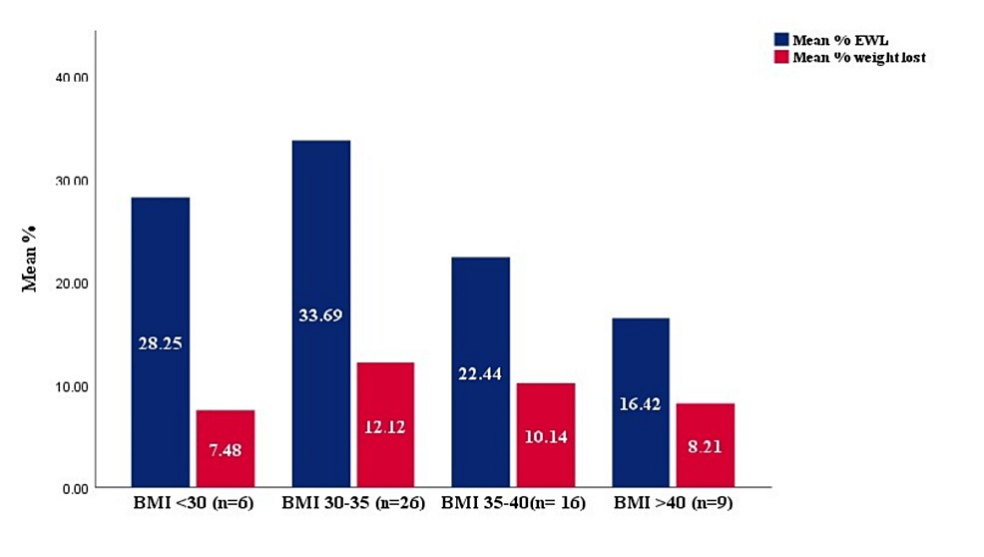
%TBWL and %EBWL based on the BMI category %TBWL: percentage total body weight loss, %EBWL: percentage excess body weight loss, BMI: body mass index

**Figure 3:**
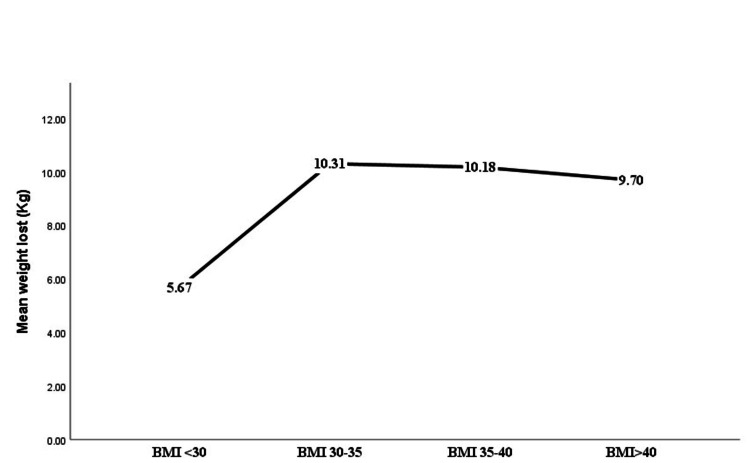
Mean TBWL based on the BMI category on intragastric balloon insertion TBWL: total body weight loss

**Figure 4:**
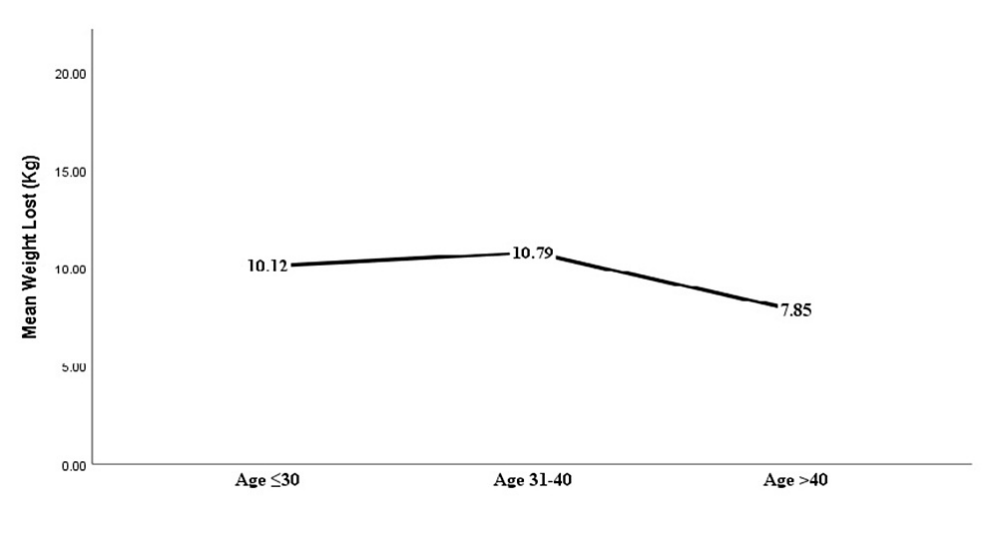
Mean TBWL based on the age group. The mean TBWL based on the age group shows that people aged less than 40 had the greatest weight loss. TBWL: total body weight loss

**Figure 5:**
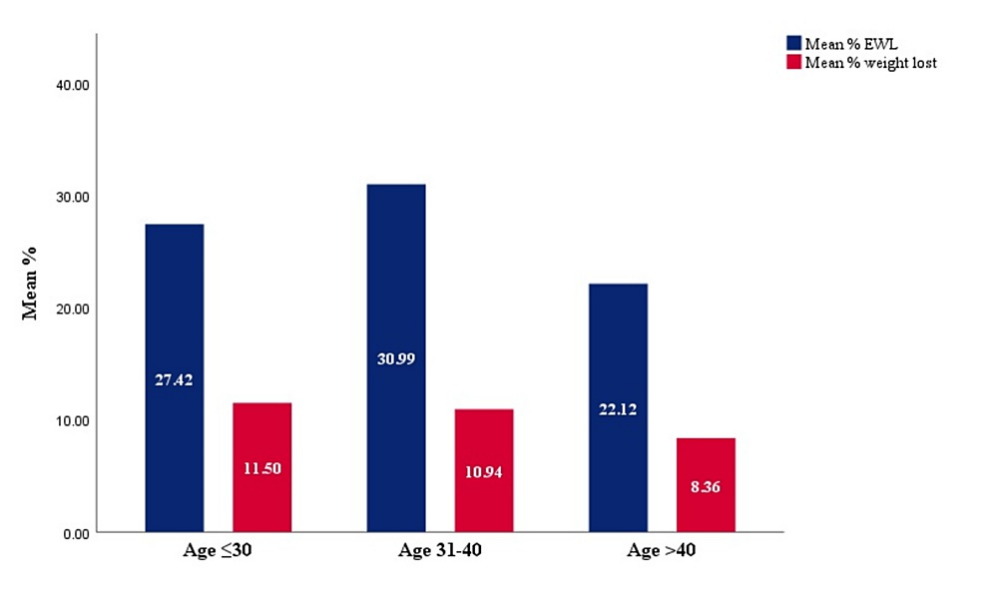
%TBWL and %EBWL according to age The %TBWL and %EBWL according to age shows that more weight loss was achieved in those <40 years of age. %TBWL: percentage total body weight loss, %EBWL: percentage excess body weight loss, BMI: body mass index

Based on the BMI, the greatest weight loss was noted in those with BMIs ranging from 30 to 35 and 36 to 40, as shown in Table 3, Figure 2, and Figure 3. The marginally significant reduction in %EBWL was seen in the patients with BMI ranging from 30 to 35 and 36 to 40 with a p-value = 0.06. On the other hand, on looking at the different age groups, the least weight reduction was noted in those aged more than 40 years with a mean TBWL of 7.85 kg. This was in contrast to those aged less than 30 and 31-40 in which the mean TBWL was 10.12 and 10.72 kg, respectively (Figure 4). Figure 5 illustrates the %TBWL and %EBWL among the age groups. The p-value for weight loss data based on age group was not statistically significant as well (Table 4).

**Table 3:** Weight loss based on the body mass index category %EBWL: percentage excess body weight loss

Group	BMI < 30 (n = 6)	BMI 30-35 (n = 26)	BMI 35-40 (n= 14)	BMI >40 (n= 10)	P-value (Kruskal- Wallis test)
Mean total body weight loss, kg	5.66 ± 5.95	10.31 ± 9.19	10.17 ± 7.11	9.70 ± 11.43	0.37
Mean %EBWL	28.2 ± 27.84	33.69 ± 20.99	22.44 ± 14.63	16.42 ± 20.55	0.06
Mean %total body weight loss	7.48 ± 6.56	12.11 ± 8.43	10.14 ± 6.52	8.21 ± 10.09	0.19

**Table 4:** Weight loss according to age

Group	Age ≤30 (n = 23)	Age 31-40 (n = 19)	Age > 40 (n = 15)	P value (Kruskal-Wallis Test)
Mean total body weight lost (kg)	10.17 ± 11.63	10.79 ± 5.30	7.8 ± 7.02	0.30
Mean %excess body weight loss	27.42 ± 24.18	30.99 ± 17.43	22.1 ± 18.99	0.31
Mean %total body weight loss	11.50 ± 10.27	10.94 ± 5.25	8.36 ± 7.27	0.40

Sixty-five patients responded to the telephone interview, which was used to assess the side effects and satisfaction of the procedure. With regards to side effects and complications, nausea, vomiting, and abdominal pain were the most common among the patients with an average duration of 2.6 ± 4.4 weeks (Table 5). Furthermore, 13 patients had severe symptoms including intractable nausea, vomiting, and abdominal pain to the extent that they did not tolerate the IGB and had it removed prematurely. Other patients were able to tolerate the transient side effects with the aid of medications provided.

**Table 5:** Frequency of adverse effects

Adverse effect	Frequency (percentage)
Nausea	60 (92%)
Vomiting	45 (69%)
Abdominal pain	42 (64%)
Heartburn	21 (32%)
Indigestion	11 (17%)
Bloating	20 (31%)

Minor complications included three ruptured balloons, two of which did not have their IGB removed on time and one migrated balloon in a patient who removed the IGB after 14 months. Major complications like gastric obstruction, gastric perforation, and bleeding ulcer were not found. Procedure-related adverse events that occur during balloon removal, including esophageal tear, pneumonia, gastrointestinal bleeding, and esophageal perforation were also not found. No mortalities occurred in this study.

Of the patients who tolerated IGB insertion, 52 responded to the telephonic interview. 26 patients (50%) of these patients were satisfied with the procedure and the overall result. Similarly, 26 patients (50%) were not satisfied with the procedure.

Nine of the patients who were not satisfied with the result went on and underwent bariatric surgery after IGB removal.

An additional observation was that 17 patients (23.9%) were *Campylobacter*-like organism (CLO)-positive, of which five patients could not tolerate the IGB. The CLO-positive patients were at a greater risk of developing non-tolerance to IGB with an OR of 2.15 (95% CI 0.39-9.28, p-value = 0.3). However, this was a statistically insignificant finding. 

## Discussion

In a retrospective study, we have substantiated the efficacy and safety of the IGB as a modality for weight loss among individuals who are overweight or obese. The IGBs used in our study were Medsil and Medicone. Almeghaiseeb et al. have reported their findings, indicating that the deployment of Medsil IGB holds no discernible distinction from bioenteric IGBs. Their investigation has shown a statistically significant reduction in the BMI and overall body weight. Specifically, the average loss in the BMI stood at 4.75, accompanied by a TBWL of 12.48 ± 5.16 kg at the point of IGB removal [15].

Over the past two decades, IGB effectiveness has been studied extensively. Our results are similar to the reported literature, which showed a significant weight loss. Courcoulas et al. in a multi-center, prospective, randomized trial found weight loss of 9.8 kg at six months, which is consistent with our results of 9.74 kg weight reduction [16]. Nevertheless, pooled data analysis of weight loss at six months from seven randomized controlled trials concluded that IGB resulted in an average weight loss of 7 kg [17].

Imaz et al. in a meta-analysis of 15 studies, which included 3,608 patients, proved the effectiveness of IGB at the time of removal at six months with the estimates of TBWL of 14.7 kg, BMI loss of 5.7 kg/m^2^, and 32.1% of excess body weight lost [18]. Another study by Dogan et al. demonstrated results close to our data; at the end of six months of balloon insertion, the mean TBWL was 12.5 ± 13 kg/m, while the BMI loss was 4.4 +/- 4.5 [19]. A pooled data analysis of four randomized controlled studies illustrated that 61.9% of patients with an IGB had 10% TBWL [17]. In our study, we showed that 52.63% of patients had lost >10% of weight with a reduction in EBWL by 27%, which is comparable to the results of the REDUCE Pivotal trial that found a reduction of 25.1%of EBWL [20].

IGB is considered a safe modality for weight reduction. Dayyeh et al. in a meta-analysis found that the most frequent side effects were pain, followed by nausea and vomiting after IGB implantation, occurring in 58.7% and 39.4%, respectively. They found that migration occurred in 1.4%, 0.1% gastric perforation, and 0.08% mortality [3]. In the analysis of Mayo Clinic’s database about the safety of Orbera IGB (Apollo, USA), Vargas et al. did not notice any case of pancreatitis, gastric perforation, or deaths among the 321 patients analyzed in this study [21]. On the other hand, pooled data from the American Society of Gastrointestinal Endoscopy (ASGE) publication in 2015 found that the mortality rate was 0.01% [3].

In comparison to bariatric surgery in which serious side effects can vary between 0.1% and 0.5% [22], we did not find any serious complications throughout the six months of IGB placement.

The American Society for Metabolic and Bariatric Surgery guidelines recommend the use of extensive education and providing the patient with psychological, nutritional, and exercise counseling through a multidisciplinary team to achieve the best results [23].

Patients who had a positive rapid urease test were at a higher risk of developing IGB intolerance with an OR of 2.15. However, due to the limited number of patients, this was statistically insignificant. Further studies are needed to establish if there is an association between these two variables.

The limitations of our study were the lack of long-term data post IGB removal in terms of weight loss and compliance with diet and exercise. Nevertheless, more studies were published recently to answer the question of the long-term efficacy of IGB over 2.5 years that showed IGB is an effective modality to reduce weight if combined with commitment to diet and exercise [24]. Other limitations include the small number of patients and the retrospective nature of the study.

To the best of our knowledge, this is the first study assessing the efficacy of IGB to be conducted within the Kingdom of Bahrain and ranks among the limited studies pursued within our region. Moreover, notwithstanding the limited patient cohort, this study shows statistically significant results in terms of weight reduction, and it shows comparable outcomes to preexisting published data, particularly pertaining to the safety profile associated with the procedure.

## Conclusions

Our data affirm the efficacy and safety of IGBs as a potent tool for inducing weight loss, particularly among patients with a BMI ranging between 30 and 35. Most patients tolerated the IGB, and the most common side effects were nausea, vomiting, and abdominal pain. Importantly, the complication rate remains notably inconspicuous, marked by the absence of serious morbidity or fatalities. Worth highlighting, this is the first study addressing IGB effectiveness and tolerance in the Kingdom of Bahrain. Nevertheless, further studies are required to establish the long-term outcomes of IGB in Bahrain.
